# Present state of the Aral Sea: diverging physical and biological characteristics of the residual basins

**DOI:** 10.1038/srep23906

**Published:** 2016-04-01

**Authors:** A. S. Izhitskiy, P. O. Zavialov, P. V. Sapozhnikov, G. B. Kirillin, H. P. Grossart, O. Y. Kalinina, A. K. Zalota, I. V. Goncharenko, A. K. Kurbaniyazov

**Affiliations:** 1 Shirshov Institute of Oceanology Russian Academy of Science, Moscow, Russian Federation; 2 Leibniz-Institute of Freshwater Ecology and Inland Fisheries, Berlin, Germany; 3 Faculty of Geography, Lomonosov Moscow State University, Moscow, Russian Federation; 4 Yasavi International Kazakh-Turkish University, Turkestan, Kazakhstan; 5 Institute of Biochemistry and Biology, Potsdam University, Germany

## Abstract

Latest data on the hydrophysical and biological state of the residual basins of the Aral Sea are presented and compared. Direct, quasi-simultaneous observations were carried out in the central part of the Western Large Aral Sea, the northern extremity of the Large Aral known as Chernyshev Bay, Lake Tshchebas, and the Small Aral Sea in October 2014. The Large Aral Sea and Lake Tshchebas transformed into hyperhaline water bodies with highly special taxocene structure. The Small Aral Sea was a relatively diverse brackish ecosystem, which was rather similar to the pre-desiccation environment. The Small Aral Sea and Lake Tshchebas exhibited a fully-mixed vertical structure, whereas the Western Large Aral Sea was strongly stratified. Our data show that during desiccation, different parts of the Aral Sea experienced different environmental conditions, resulting in qualitative and quantitative differences in the physical and biological regimes among the different residual basins.

The Aral Sea (Aral) is a terminal salt lake in western Central Asia situated at the border between Uzbekistan and Kazakhstan. In the mid-20^th^ century the lake was ranked as the fourth largest inland water body with a surface area of over 66,000 km^2^, a total volume of about 1,070 km^3^, and a maximum depth of 66 m. The Aral Sea is fed by two rivers, the Amu Darya and Syr Daria, delivering 56 km^3^ of freshwater per year on average^1^. At this time, the lake level was stable, varying only slightly and remained around 53 m a.s.l. during decades. However, geoscientists established the existence of several regressions and transgressions of the water level in the paleo- perspective over the last 2000 years^2^. In the early 1960s, the freshwater inflow into the Aral Sea started to decrease and practically ceased by 1980 because of agricultural diversions and unsustainable use of water resources in the river basins. This resulted in drastic changes in the Aral’s water balance, and led to a rapid decrease of the lake’s volume^3^. In 1989, the northernmost part of the lake, known as the Small Aral Sea, separated from the principal water body. Subsequently, the southern part, referred to as the Large Aral Sea, is divided into two parts, the Eastern Large Aral and the Western Large Aral, maintaining intermittent connection through a narrow channel in the north. Geomorphological erosion in the channel connecting the two lobes was an important process affected the interbasin exchange and evolution of the water masses^4^. Desiccation of the Aral Sea continued intensively throughout the last decade. As reported by NASA and widely commented in mass media, the eastern lobe of the Large Aral Sea dried up completely in the summer of 2014 (but then partly filled again in the spring of 2015). For the Large Aral Sea, separation of its northernmost portion called Chernyshev Bay from the rest of the basin is imminent. The Small Aral Sea level has eventually stabilized as a result of a man-made dam built in 2005, trapping all of the Syr Daria discharges within the Small Sea (and thus deteriorating the situation of the Large Aral Sea). In addition, Lake Tshchebas, formerly a large bay of the Aral Sea, has evolved into a separate lake with relatively stable boundaries. The critical morphological changes and progressing salinization resulted in profound alteration of biological system of the sea. The replacement of freshwater and brackishwater biological communities by broadly euryhaline species of marine and freshwater origin occurred. In this connection, the structure and diversity of biological communities has been reduced while their productivity increased. Accordingly, the present-day Aral Sea can be considered as a system of separate water bodies with a common origin but different fates, i.e. very different physical, chemical, and biological features.

The Aral Sea environmental crisis has resulted in strong negative impacts on regional climate, ecological conditions^5^, and quality of life of the local population^6^. Thus, monitoring the ecological and environmental changes in the Aral Sea is necessary to evaluate the success of the restoration project in the Small Aral Sea and to plan future steps of mitigation in the southern part of the Aral. Considering the continuing salinization of the Large Aral, the potential of commercial-scale brine shrimp (*Artemia*) egg production needs further research^7^. The ongoing changes in the Aral also allow for the opportunities to study the evolution of a large inland water body affected by anthropogenic intervention through diversions of the river runoff and the related ecological consequences.

The desiccation of the Aral Sea is perhaps the most illustrative example of numerous lakes of the World which suffered shallowing and degradation during the last century. The most well-known cases are Lake Chad which has shrunk to about a twentieth of its pre-desiccation size and fragmented into separate residual water bodies, Lake Lop Nor which completely dried up in the early 1970s, and the Dead Sea whose level drops steadily at about 1 m/year and present salinity is about 340 g/l^8^. Recent studies reveal negative trends in water budgets of many lakes in arid and semi-arid regions due to both climatic and anthropogenic influences^9^. However, the Aral Sea has been subject to the most intense transformations over relatively short period of time, and as such it represents an elucidating “extreme model” of processes that take place elsewhere, although manifested to a less dramatic extent.

The objective of this study is to investigate the present hydrological and biological state of the residual water bodies of the Aral Sea. The study was a part of the long-term research and monitoring program launched in 2002 by the Shirshov Institute of Oceanology of the Russian Academy of Sciences. Field observations were conducted in collaboration with a number of institutions in Russia and Central Asia’s states of Uzbekistan and Kazakhstan. The previous fifteen surveys of the program between 2002 and 2013 were mainly limited to the Large Aral Sea^10^,^11^,^12^, and did not include the Small Aral Sea or Lake Tshchebas. In view of the ongoing profound changes of the Aral ecosystem, we expanded the area of observations (Fig. 1) and examined the state of all 3 major residual parts of the Aral Sea in 2 surveys in fall 2014. Such an approach allowed for a qualitative and quantitative comparison of the hydrographic and biological properties among the three separate water bodies of the Aral Sea. We also discuss main processes and factors affecting the state of the residual basins.

## Results

### Hydrology

#### Large Aral Sea

The thermal vertical structure of the Western Large Aral’s central part exhibited a “two-layered” pattern (Fig. 2). The upper mixed layer at the station A (total depth 32.6 m) extended down to approximately 22 m with a salinity of about 115 g/kg and a temperature of about 14.5 °C. From 22 m down to the bottom, there was a stratified layer with a steep thermocline and a pronounced increase in salinity. The vertical temperature gradient in this layer was as high as 0.9 °C/m, while salinity and temperature near the bottom reached 121.5 g/kg and 5 °C, respectively. It should be pointed out that the presence of a strong vertical stratification was the distinctive feature of the western basin of the Large Aral since the beginning of 2000^10^,^13^. Increase in salinity in the bottom layer was typically accompanied by a temperature inversion. This pattern is caused by water exchange between the eastern and western basins of the Large Aral. Salinization of the broad shallow eastern basin was more intense since the Large Aral’s separation into two parts. However, the eastern and western basins remained connected through a narrow but relatively deep strait in the northern part of the Large Aral. Much saltier and denser water from the east filled the bottom layer of the western basin and led to very stable density stratification. The TS-analysis based on CTD-measurements obtained in autumn 2002 revealed that 9 to 11% of water in the bottom layer of the western basin had originated from the eastern basin. Direct measurements in 2004 yielded an estimated inflow of about 1500 m^3^/s^10^. However, the impact of this inter-basin exchange on the formation of a vertical stratification in the western lobe has practically ceased due to the rapid decrease in the volume of the eastern basin during the last 5 years. The latter resulted in a gradual decrease in salinity difference between surface and bottom layers, and the magnitude of temperature inversion. Thus, according to direct measurements in 2010, salinity of the bottom water at station A was 132 g/kg, and the magnitude of temperature inversion in the bottom layer was 5 °C^12^. In contrast, the latest observations in 2014 showed a decrease in bottom salinity and the disappearance of temperature inversion.

Hydrographic measurements conducted at the station C1, the deepest part of the Chernyshev Bay, revealed a complex vertical thermal structure of the water column (Fig. 2). The upper mixed water layer extended down to 5 m depth with a temperature of 11.2 °C. Below the upper layer, between 5 and 6.3 m depth, an extremely steep temperature inversion occurred, constituting a jump of more than 12 °C across a layer of only 1.3 m thickness. From here the temperature decreased to 14.9 °C at the bottom. Temperature values of about 24 °C, measured immediately below the upper mixed layer, were likely the result of intense summer warming of the water column, which was particularly strong in the shallow eastern basin. For technical reasons, we were unable to collect any salinity data from the Chernyshev Bay. However, to achieve a density profile with vertical stability, the observed temperature inversion of nearly 12 °C must have been accompanied by a commensurately abrupt increase in salinity of 3–4 g/kg or more. Such a saltier and warmer water mass underlying the upper mixed layer in the Chernyshev Bay most likely originated from the eastern basin. This suggests that while the scale of the eastern basin water inflow decreased in the past years due to massive shrinking of the eastern lobe, the Chernyshev Bay is still largely influenced by it. This influence may have even increased in parallel to the progressive shoaling of the sill between the Chernyshev Bay and the rest of the western basin, which increasingly traps the incoming eastern basin water within the Chernyshev Bay.

The influence of the eastern basin water inflow in the Chernyshev Bay is also confirmed by direct measurements carried out at station C2 located about 1.5 km from the strait mouth (Fig. 2). Despite the relatively shallow depth (about 5 m) in the surrounding area, the water column was stratified with a salinity of 131.7 g/kg at the surface and 133.8 g/kg near the bottom, which is more than 12 g/kg higher than the salinity maximum of the bottom layer in the central part of the western basin.

#### Small Aral Sea

Hydrological observations carried out at the two stations in the Shevchenko Bay showed that the water column in this part of the Small Sea was vertically mixed with a salinity of about 11.1 g/kg (Fig. 2). Water temperature at the deeper station M1 was 11.3 °C, while temperature at the station M2, which is situated closer to the shoreline, varied between 9.3 and 9.7 °C. According to historical data, the annual average salinity in the Shevchenko Bay during 1956–1960 was 9.5 g/kg^1^. Thus, the present-day salinity of the Small Aral is 1.6 g/kg higher than before the desiccation onset - although the water level (approximately 42.5 m a.s.l.) is around 10.5 m lower than in 1960. The present day volume of the Small Aral is estimated as 27.5 km^3^.

#### Lake Tshchebas

According to our field data, water of Lake Tshchebas was almost uniformly mixed from surface to bottom (Fig. 2). Measured salinity was 91.9 g/kg at the surface and 92.1 g/kg at the bottom, while temperature varied from 10.3 °C at the surface to 10.1 °C at the bottom. Observed quasi-homogeneous structure of the water column is likely a result of wind mixing of the shallow water body, possibly amplified by autumn convection. The present day’s volume of Lake Tshchebas is estimated as 1.9 km^3^. The overall water level of Lake Tshchebas dropped by ca. 22 m since 1960, which is more than 8 m less than in the western Large Aral. Over the last years, however, the water level of Lake Tshchebas remained relatively stable. The details of Lake Tshchebas’ water budget remain unknown. One source of water supporting the stability of the lake’s water level is episodic releases of water from the Small Aral through the Kokaral Dike, which partly ends up in Lake Tshchebas, as it can be seen in the satellite imagery (see Supplementary Fig. S1). Another potential source is groundwater discharges. Some groundwater seepage have been observed at the shores of Lake Tshchebas. However, no estimates of the volume of groundwater discharges are available to date.

### Eubacteria and Archaea community structure

Analysis of the eubacterial community structure revealed clear differences between the 3 different residual lake basins (Fig. 3a,b). Whereas eubacterial communities of the very slightly stratified Lake Tshchebas and Small Aral did not differ much between surface and bottom samples, they greatly differed for the stratified Large Aral station C1. Cluster analysis of DGGE banding patterns reveals that eubacterial communities from the bottom of the Large Aral were most distinct, whereas those from the surface of the Large Aral were more similar to the surface and bottom communities of Lake Tshchebas than to those of the Small Aral. The low similarity in eubacterial communities of the Small Aral reflects its rather brackish nature, whereas Lake Tshchebas and the Large Aral are characterized by hyperhaline conditions (see above). Thus, bacterial communities reflect both hydrophysical and chemical conditions in the 3 residual basins of Aral Sea. Moreover, DGGE analysis of Archaea communities was performed (see Supplementary Fig. S2). In contrast to the Eubacteria, Archaea communities reflect less the differences in physical and chemical conditions between the different studied basins. Although clear differences between surface and bottom communities in each of the basins were observed, surface and bottom communities of the strongly stratified Chernyshev Bay were more similar to each other, but differed in the well mixed Small Aral. In addition, surface communities of Lake Tshchebas and Chernyshev Bay were almost the same. However, all bottom communities were different indicating differences in salinity and origin of the water body as major controlling factors.

### Biodiversity of benthic organisms

A broad spectrum of biotic communities has been studied in the water bodies of the former Aral Sea^13^,^14^. In this study, however, we focused solely on the benthic diatom taxocene as the most diverse and ecologically informative group of organisms for our comparative study of the three Aral basins.

Overall, 171 species and subspecies taxa of diatoms have been found in the three studied basins of the Aral Sea. Therein, the benthic flora consisted of 150 taxa in the Small Aral Sea (see Supplementary Figs S3–S5), 29 taxa in Lake Tshchebas (Supplementary Figs S6 and S7), whereas in the hyperhaline Western Large Aral (Supplementary Figs S8 and S9), only 7 species were found (*Amphora holsaticoides, Halamphora acutiuscula, Halamphora cymbifera, Halamphora normanii, Halamphora subholsatica, Navicula cryptotenella*, and *Nitzschia liebetruthii*). Those can be considered as “exceptionally broadly euryhalobic” species which thrive equally well in the Small Aral (salinity 10.7 g/kg) and in the Large Aral (salinity 115.4 g/kg).

Another 5 species (*Halamphora coffeaeformis, Halamphora tumida, Fallacia tenera*, *Nitzschia sigmaformis,* and *Navicula digitoradiata*) have only been found in the Large and Small Aral, but not in Lake Tshchebas. *N. digitoradiata* was found in similar biotopes of the Large and Small Aral (at 8.5 m and 12 m depths, respectively), but not in Lake Tshchebas. Nevertheless, these five species can be considered as broadly euryhalobic.

In the Small Aral and Lake Tshchebas, 9 species were found in salinity ranging from 10.7 to 91.9 g/kg: *Cocconeis placentula* var. *euglypta, Entomoneis paludosa, Gyrosigma fenestratum, Mastogloia pumila, Nitzschia fonticola, Navicula rhamosissima, Navicula rossii, Rhopalodia musculus,* and *Tryblionella apiculata*. Our earlier studies have shown that the first five of these species also occur in the Large Aral at salinities ca. 100–110 g/kg^13^,^14^. It is possible that in October 2014 dispersal of these species into the Large Aral has not taken place, presumably because of progressive changes in physical and chemical conditions. Possibly, a greater variety and number of studied biotopes is needed to also detect them in the Large Aral. Finally, another 3 species were only found in Lake Tshchebas and in the Large Aral; they can be considered as “relatively ultrahalobic” and consist of *Amphora pusio, Halamphora dusenii,* and *Navicula* sp. 1.

The Sorensen similarity index (K) for the diatom flora of the studied residual water bodies was 23.4% on average, indicating that species composition in each basin significantly differed from that in the other basins. However, the flora of the Large Aral was more similar to the flora of Lake Tshchebas (K = 38.4%). The species assemblage of the Small Aral significantly differed from that of Lake Tshchebas (K = 17.9%) and differed even more from the Large Aral (K = 13.9%).

Diatomic taxocene structures and species diversity of different biotopes were analyzed using the Bray–Curtis dissimilarity index. Specific groupings of species developed in each of the three basins differing in dominating species, mass, and frequency of occurrence (Fig. 4a). The Small Aral’s taxocene had the lowest intra-group similarity (22.4%). This could be due to the fact that the taxocene which was found at 12 m depth was significantly different from that in the shallow water (0–30 cm depth) communities. A community dominated by *Caloneis aemula* and *Campylodiscus echeneis* existed on muddy sediments at 12 m depth. In the shallow depths with muddy sand sediments, *Navicula phyllepta, Mastogloia smithii, Mastogloia pusilla, Mastogloia lanceolata, Halamphora corallineana, H. tumida, Planothidium engelbrechtii, Parlibellus cruciculoides, Tryblionella hungarica, T. apiculata* and *Navicula salinarum* dominated. On the other hand, the Large Aral community were split into two subcommunities based on the Bray–Curtis dissimilarity index (Fig. 4b). One subcommunity developed on the surface of hard substrates such as stones and shells in shallow water, on the sand of the swash zone in Chernyshev Bay as well as on black mud sediments at 8.5 m depth. This subcommunity was characterized by mostly sedentary diatoms: *Halamphora* (*H. normanii, H. cymbifera*) that were semi-attached to the mineral substrate. The second subcommunity was dominated by motile species such as *Nitzschia* (*N. sigmaformis, N. communis*) which inhabited silt deposits on the surface of clay sediments and salt sand sediments beneath the waterline of the shallow shore.

## Discussion

Direct quasi-simultaneous measurements conducted in October 2014 revealed that each of the remaining Aral Sea basins acquired different hydrologic features (Supplementary Table S6). Evaluating the differences between the basins, one should keep in mind that the internal variability within each of the basins could not be fully assessed in this sampling campaign because of limited spatial coverage. However, to the extent we can judge based on the collected evidence, the internal differences between different sites within each basin were generally much smaller than those between the regions: so, e.g. the variance analysis for the salinity, as a major characteristic of the physical and chemical regime of the residual basins, suggests that the Chernyshev Bay has different waters than the Large Aral with 98% confidence. Lake Tshchebas and the Small Aral are clearly different from each other and from all other basins at the confidence level >99.9.

Although of common origin, the residual basins were influenced by various factors, leading to qualitative and quantitative differences in their hydrophysics, chemistry, and biology. Obviously, presence/absence of freshwater inflow was the principal factor. The Large Aral and Lake Tshchebas transformed into hyperhaline water bodies due to a severe lack of river water inflow. In contrast, the Small Aral was regularly fed by the Syr Daria river and thus retained a brackish nature close to the pre-desiccation salinity level. On the other hand, other factors determined the vertical structure of the water column. The relative shallowness of the Small Aral and especially of Lake Tshchebas facilitated wind-induced mixing, and consequently both basins were nearly fully mixed. Other potential mechanisms of vertical mixing included haline convection following intense evaporation in summer, and thermal convection due to cooling in winter. At the same time, the Western Large Aral was stratified in its central part and in Chernyshev Bay. Apparently, this resulted from advection of saltier water from the eastern basin through the connecting strait, as seen during the last decade. Although the intensity of vertical stratification in the central part of the basin gradually decreased in connection with the shrinkage of the eastern part of the Large Aral and the reduction of the inter-basin exchange, the Chernyshev Bay still remained exposed to this inter-basin water exchange due to both the proximity of the strait outlet and the topography of the Chernyshev Bay. Topohgraphic isolation will increase as the drop in water level progresses. Large differences in vertical density structure, mixing conditions and salinity regimes resulted in very diverse ecosystems as demonstrated by the large differences in benthic and bacterial communities in the residual lake basins. The brackish Small Aral has apparently retained all the major hydrophysical features of the parental Aral Sea, and is regularly mixed down to the bottom on seasonal time scales, as demonstrated by the homogeneous vertical temperature and salt distribution. In all basins, temperature profiles revealed a nearly homogeneous vertical distribution in the upper part of the water column (at least 5 m depth). However, the upper homogeneous layer of the Large Aral extends down to 22 m depth. This indicates intensive mixing in the upper water column of the different lake basins despite their appreciable vertical salinity gradients (0.2 g/kg per 4 m in Lake Tshchebas and as much as 2.1 g/kg per 5 m in Chernyshev Bay). While wind may be regarded as the main force of surface mixing in shallow and weakly stratified waters of the Small Aral and Lake Tshchebas, an additional mechanism should be responsible for mixing the upper 20 m of the Large Aral and the strongly salt-stratified Chernyshev Bay. Pronounced lateral salinity gradients (surface salinity difference of 16.3 g/kg between the central part of the Large Aral and Chernyshev Bay) suggest an intense lake-wide density circulation, which may affect mixing. The precise circulation features, however, are not known, and should involve complex interactions between water masses with different temperatures and salinities. This includes plunging underflows, double diffusion, and contraction by mixing (cabbeling), mechanism which are known to play an important role in other saline and freshwater lakes^15^,^16^,^17^. The deep areas of the salt basins (beneath 22 m in Large Aral and beneath 6 m in the Chernyshev Bay) are thermally stratified, whereas it is still possible that the Large Aral can be mixed completely during cold winters, which are characteristic of an arid climate. Chernyshev Bay in turn has a very strong halocline between 6 and 8 m depth marked by the inverse (unstable) thermal stratification with a local temperature maximum of ~7m, resembling the situation typical for meromictic (never mixed to the bottom) salt lakes^18^. The warm layer was most likely created by the intrusion of warm salt water from the eastern lobe of the large Aral, which has already been dried up in the preceding summer, i.e. the thermal inversion should have been supported for several seasons, being a strong indication of a monimolimnion (a non-mixed layer without any exchange with the upper waters except for slow diffusion processes) beneath.

These lake features are well represented by differences in eubacterial and diatom microphtobenthos communities of the studied basins. The eubacterial communities clearly reflect hydrophysical properties of the 3 basins, in particular the stable stratification in the Chernyshev Bay resulted in 2 very distinct communities (Fig. 4a,b). Moreover, similarity between the eubacterial communities of different basins was determined by lake connectivity (water inflow) and level of salinity. Whereas the eubacterial communities (surface and bottom) of Lake Tshchebas were relatively similar (60%) to that in the surface of the Large Aral, similarity with those of the Small Aral was much lower (<40%). Our analysis indicates higher species richness for the brackish Small Aral in comparison with the more saline Lake Tshchebas and the Large Aral, and in particular the very saline bottom water of the Chernyshev Bay.

A similar pattern can be seen for the diatom microphytobenthos, which showed high species richness and diversity for the Small Aral in comparison to Lake Tshchebas and the Large Aral. Among the 3 basins, the average similarity of the diatom taxocene flora was low (K = 23.4%). Thereby, the Large Aral and Lake Tshchebas were more similar to each other (K = 38.4%) than to the Small Aral (K = 13.9% and K = 17.9%, respectively). Groupings of mass and dominating species were different for each basin. Taxocene structures in the brackish Small Aral were most diverse and the dominating species had the lowest significance in the ecosystem. The taxocene of the Large Aral and Lake Tshchebas could be described as extreme (euryhalobic), whereas this was not the case for the brackish Small Aral. The different timing in separation of the three basins from the original Aral Sea led to the development of taxocenes with broadly euryhalobic species having a significant effect on the overall taxocene structure. In contrast, in the brackish Small Aral, a significant proportion of the mass species were meso- and euhalobic.

As an immediate consequence of the newly formed meromixis, a strong alteration of the oxygen regime can be expected in the Chernyshev Bay and, probably, in the Large Aral, with manifold effects on the ecosystem level. Isolation of deep waters from the atmosphere together with low rates of photosynthesis will inevitably produce deep anoxia, which is already the case in some areas of these basins^13^. The high amount of organic matter deposited over the centuries in the Aral Sea provides a rich source of nutrients for anoxic microorganisms favoring methanogenesis In the Small Aral, the water column remains well-oxygenated down to the bottom throughout most of the year and development of anoxia is unlikely. Hence, a continuous divergence of the biological communities can be expected in the newly divided basins, whereby some of them may potentially turn into significant methane sources. Undoubtedly, the hydrology and the ecology of the newly formed residual lakes of the former Aral Sea will remain much more variable in the following decades than those of ‘normal’ inland water bodies; however, eventual stabilization of their water budgets will slow down the external stresses on the lakes and will provide a unique opportunity to study the development of new aquatic systems.

## Methods

### Sites and Sampling strategy

**Figure 1:**
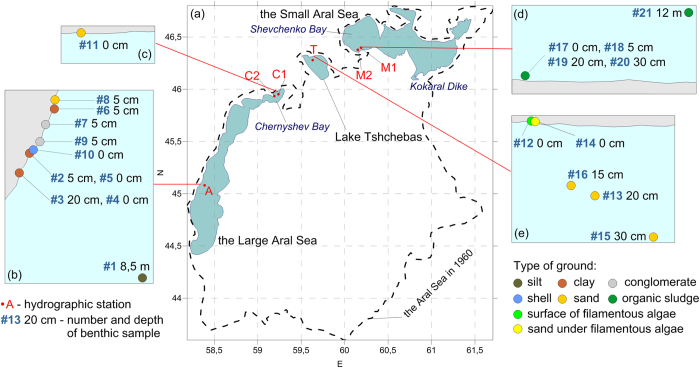
Map of the three residual basins of the Aral Sea and positions of the hydrographic stations (**a**). Schemes of microphytobenthos material sampling in the Large Aral Sea (**b**), Chernyshev Bay (**c**), the Small Aral Sea (**d**) and Lake Tshchebas (**e**). Map and schemes were generated using Surfer 12 software, http://www.goldensoftware.com/.

**Figure 2:**
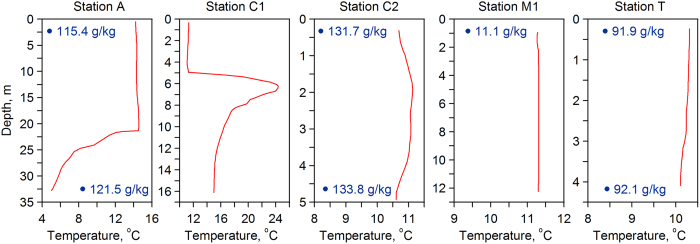
Vertical profiles of temperature (red line) at the stations A, C1, C2, M1 and T. Salinity values of surface and bottom water layers are shown in numbers.

**Figure 3:**
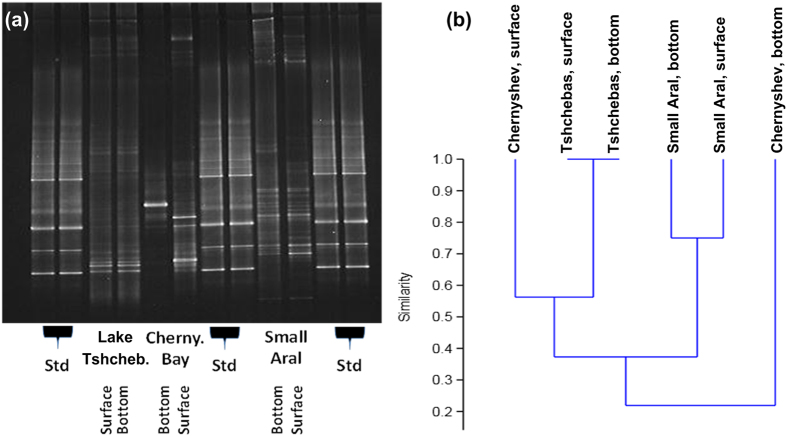
(**a**) DGGE bands of eubacterial communities in surface and bottom samples of the 3 sampled Aral basins: Lake Tshchebas, Chernyshev Bay (Large Aral Sea), Small Aral Sea; Std: standard. (**b**) Cluster analysis of DGGE banding pattern.

**Figure 4:**
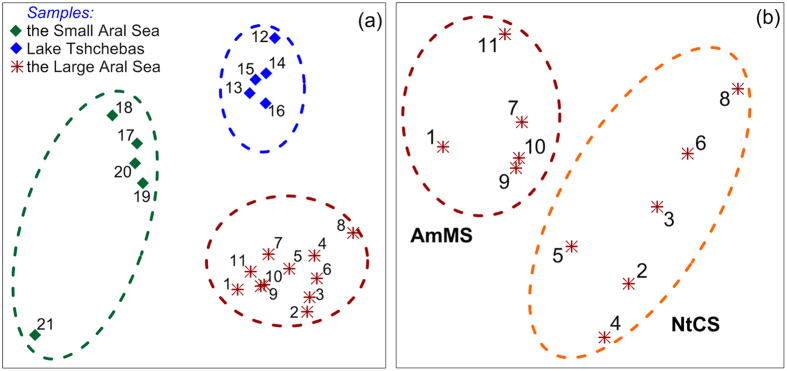
(**a**) Taxocenic grouping of the diatom microphytobenthos in the 3 Aral Sea basins. (**b**) Two subcommunities in the taxocene grouping of benthic diatoms in the Western Basin of the Large Aral Sea: AmMS – subcommunity dominated by amphoroides on mineralized substrate, NtCS – subcommunity dominated by nitzschioides on clay substrate. Numbers show information about samples according to Supplementary Table S1.

The western basin of the Large Aral Sea is situated in a trench with a steep bottom slope at the western side of the former Aral Sea where the maximum depth was approximately 35 m in 2014 (Fig. 1). The Chernyshev Bay is located in the northern extremity of the western Large Aral. Its deepest central part (maximum depth about 17 m) is connected with the main lobe of the western basin through the shrinking shallow area, where the erosional channel of density current is observed by remote sensing. The Small Aral (also known as the North Aral Sea) is a brackish water body in the north of the former Aral Sea fed by the Syr Daria river. The Small Aral was connected with the southern part through the Berg Strait and the Auzi-Kokaral Strait until 1988–1989, when the Large and the Small Aral separated from each other. To prevent further shrinking of the Small Aral, the Kokaral Dike was built in 2005 in the Berg Strait. This retained the Syr Daria river discharge within the Small Aral and facilitated an increase in the lake’s water level. Lake Tshchebas, a former bay of the Aral Sea, is situated in the north-western part of the region, bounded by the Ustyurt plateаu. It is a shallow water body with a maximum depth of ca. 8.5 m. Lake Tshchebas separated from the Large Aral in 2005–2006, during an intense sea level drop following completion of the dam in the Berg Strait. By 2014, this lake became a rather peculiar hyperhaline water body.

The presented data were obtained during the two field surveys in the fall season of 2014. The first survey was conducted in the Uzbekistan, deep western part of the Large Aral during 3 days from 6 to 8 October, 2014. The second survey took place on October 20–26, 2014 and covered the Aral Sea basins within the Kazakh territory. The research areas of these two expeditions encompassed the 3 principal water bodies situated within the former boundaries of the Aral Sea.

Direct hydrophysical and biological measurements were carried out using two inflatable motorboats at six stations (Fig. 1). Station A was positioned in the central part of the western basin of the Large Aral with a total depth of 34 m. Stations C1 and C2 were situated in the Chernyshev Bay of the Large Aral, with a total depth of 16 and 5 m depth, respectively. Stations M1 and M2 were 12 m and 5 m deep, respectively, in the Shevchenko Bay, the western part of the Small Aral Sea. Finally, Station T was positioned in the northern part of Lake Tshchebas with a total depth of 4 m. The data we collected in these parts of the former Aral Sea were the first in a long period of time (except the western Large Aral, where measurements have been taken annually over the last 13 years). The most recent *in situ* observations date back to 2002 for the Chernyshev Bay^19^, the late 1990s for the Small Sea^20^,^21^, and the late 1980s for Lake Tshchebas (i.e., the Tshchebas Bay at the time)^1^. Undoubtedly the physical, chemical, and biological regimes of these water bodies have changed drastically since then.

The sampling locations in this study as shown in Fig. 1 were determined by logistic restrictions. The Aral Sea is bounded from the west by rocky cliffs of the Ustyurt Plateau, and the newly dry lake bottom is usually impassable even for off-road vehicles. In consequence, it was imperative to use the very few sites in the region where it was feasible to come close to the water and put boats afloat. However, we presume that conditions at the selected locations were representative of the respective lacustrine regions, as the stations were situated in open, central parts of the water bodies. To evaluate the local spatial variability in the Chernyshev Bay and the Shevchenko Bay, we collected material at two stations in each basin, one in the near-shore area of 4–5 m depth, and the other one farther offshore of about 12–16 m depth. In the shallow Lake Tshchebas where maximum depth did not exceed 5 m, we occupied only one station, as well as in the central part of the western Large Sea. For the latter, the location of station A has been shown to be representative of the entire basin^13^.

We also note that the measurements and sampling at all sites were done within a relatively short time between October 6 and October 26, 2014. Generally, temporal variability of the Aral Sea is dominated by intense seasonal cycling and strong interannual trends. Accordingly, conditions during 21 days can be considered nearly constant. For example, the average change of sea surface temperature over this period was only 4 °C, compared with the annual temperature variation exceeding 28 °C^13^. In addition, the meteorological and weather conditions were stable throughout the survey. Cloudy weather with daytime air temperature about 5–7 °C and about 0 °C at nighttime accompanied by wind gusts up to 10 m/s speed persisted throughout the observation period. In this sense, our observations in different basins can be considered quasi-simultaneous.

### Sampling and analytical methods

*In situ* measurements at each station included surface-to-bottom CTD-profiling using a *YSI 6600* instrument. True salinities of water samples collected by a 5-liter Niskin bottle at two horizons (0 m and bottom) were obtained in laboratory analyses through the dry residue method.

The surface and bottom water eubacterial communities from the following locations were analyzed: the Large Aral Sea (Chernyshev Bay), Lake Tshchebas, and the Small Aral Sea (Shevchenko Bay). Circa 100–150 ml sample water was filtered onto 0.2 μm Nuclepore polycarbonate filters, fixed with pure ethanol, and stored at 4  °C until analysis in the lab. Extraction of genomic DNA was performed, using a standard protocol with phenol/chloroform/isoamylalcohol, SDS, polyvinylpyrrolidone, and zirconium beads as previously described in detail^22^. For denaturing gradient gel electrophoresis (DGGE) a 550 bp fragment of the 16S rRNA gene was amplified using the primer pair 341f and 907r (5′–CCT ACG GGA GGCAGC AG–3′ and 5′–CCG TCA ATT CMT TTG AGTTT–3′). At the 5′-end of the primer 341f, an additional 40 bp GC-rich nucleotide sequence (GCclamp) was added to stabilize migration of the DNA fragment in the DGGE. DGGE has been performed in a 7% (v/v) polyacrylamide gel with a denaturing gradient from 40 to 70% of urea and formamide^23^. For Archaea, a nested PCR approach was used. In the first round, a PCR protocol with primer set 21F–958R was used, which did not result in false positive bands. In the second round, the Parch519f–Arch915rPCR primer set was used^24^. Cluster analysis of DGGE bands has been performed by using the Phoretix 1D Advanced and Phoretix 1D Database software (Phoretix International, Newcastle upon Tyne, United Kingdom).

Furthermore, microphytobenthos material was collected from the upper sediment layer by inserting a plastic container (1 cm in diameter) 3–4 mm into the top layer of sediments and scraping three 15 cm long strips. To collect overgrowth from stones, a 3–5 cm long strip was scraped off. In addition, mollusk (*Cerastoderma isthmicum*) shells were collected from the Large Aral for epilithon analysis. Overall, 21 samples were collected for the comparison of microphytobenthos at the three basins (see Supplementary Table S1). The samples were taken at depth from 0 to 12 m from multiple locations around the respective Stations shown in Fig. 1. All samples were preserved in 2.5% formaldehyde solution. Diatomic taxocene structure of the different biotopes was analyzed using the Bray–Curtis dissimilarity index^25^ and the Sorensen similarity index^26^. Multidimensional scaling (MDS) method using Primer-6 software package^27^ was applied to obtain the MDS-diagrams showing taxocenic grouping of the diatom microphytobenthos. Hierarchical species collections that characterize community groupings for each basin were obtained using SIMPLER procedure (Primer-6 software package) (see Supplementary Tables S2–S4). Diversity (Shannon-Wiener index, (H‘)), evenness Pielou’s evenness index (J’))^28^ and the probability of interspecific encounter (PIE - Gini–Simpson index^29^) were calculated for taxocenes found in different studied habitats (Supplementary Table S5).

## Additional Information

**How to cite this article**: Izhitskiy, A. S. *et al.* Present state of the Aral Sea: diverging physical and biological characteristics of the residual basins. *Sci. Rep.* **6**, 23906; doi: 10.1038/srep23906 (2016).

## Supplementary Material

Supplementary Information
