# Supplementary material for: Present state of the Aral Sea: diverging physical and biological characteristics of the residual basins
Source: Sci Rep. 2016 Apr 1;6:23906. doi: 10.1038/srep23906 (PMC4817148; doi:10.1038/srep23906)
Supplement: Supplementary Information [file srep23906-s1.pdf]

Supplementary Information

PRESENT STATE OF THE ARAL SEA: DIVERGING PHYSICAL AND BIOLOGICAL CHARACTERISTICS OF THE RESIDUAL BASINS

A.S.Izhitskiy*[1], P.O. Zavialov[1], P.V. Sapozhnikov[1], G.B. Kirillin[2], H.P. Grossart[2,5], O.Y.Kalinina[1,3], A.K. Zalota[1], I.V. Goncharenko[1], A.K. Kurbaniyazov[4]

[1]Shirshov Institute of Oceanology Russian Academy of Science, Moscow, Russian Federation

[2]Leibniz-Institute of Freshwater Ecology and Inland Fisheries, Berlin, Germany

[3]Faculty of Geography,Lomonosov Moscow State University, Moscow, Russian Federation

[4]Yasavi International Kazakh-Turkish University, Turkestan, Kazakhstan

[5]Institute of Biochemistry and Biology, Potsdam University, Germany

Supplementary Figures

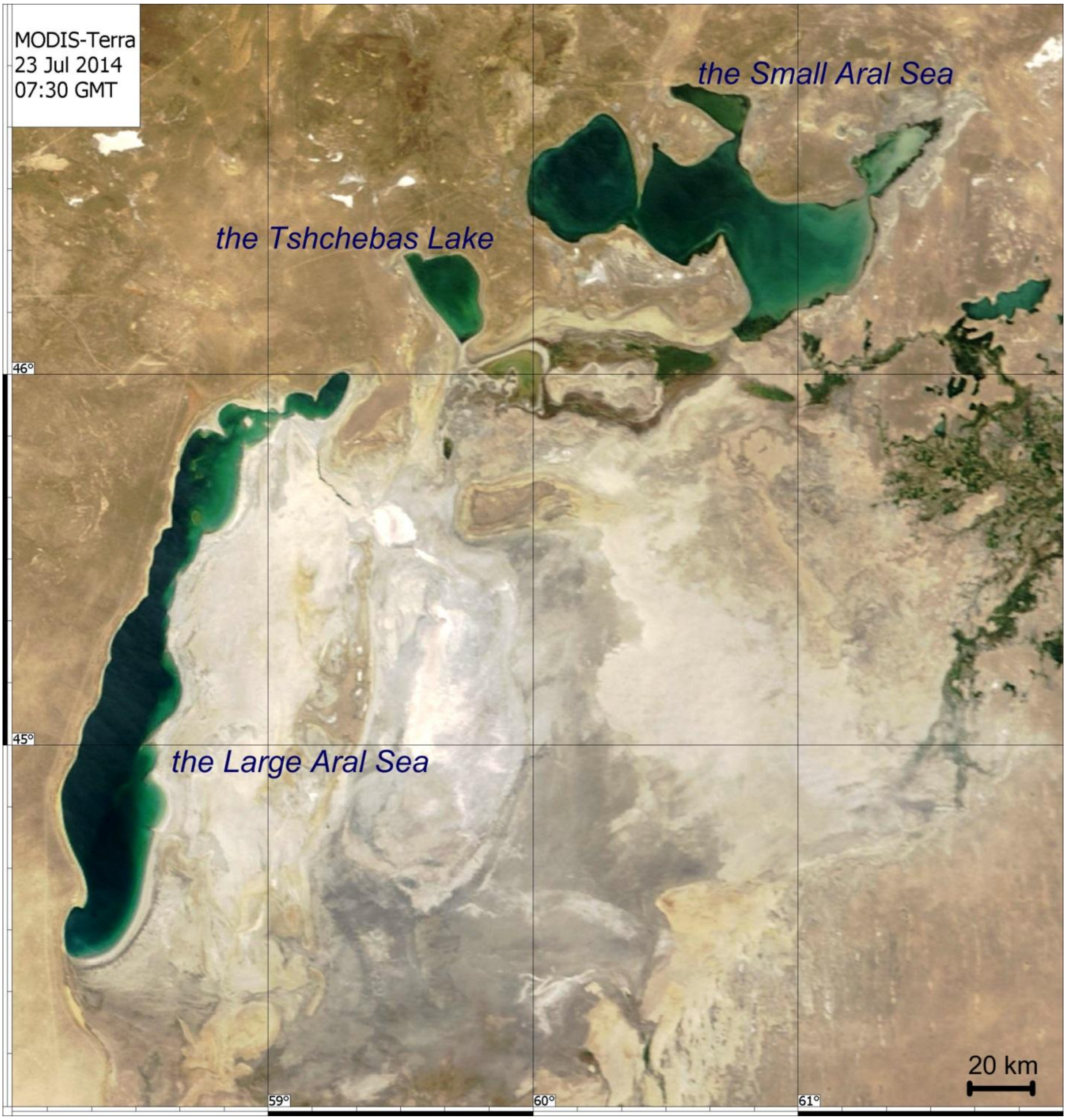

Supplementary Fig. S1. MODIS-Terra satellite image of the Aral Sea. Episodic release of water from the Small Aral through the Kokaral Dike towards Lake Tshchebas is observed. Figure was created with Nasa Worldview (https://earthdata.nasa.gov/labs/worldview/) and modified with Surfer 12 software (http://www.goldensoftware.com/).

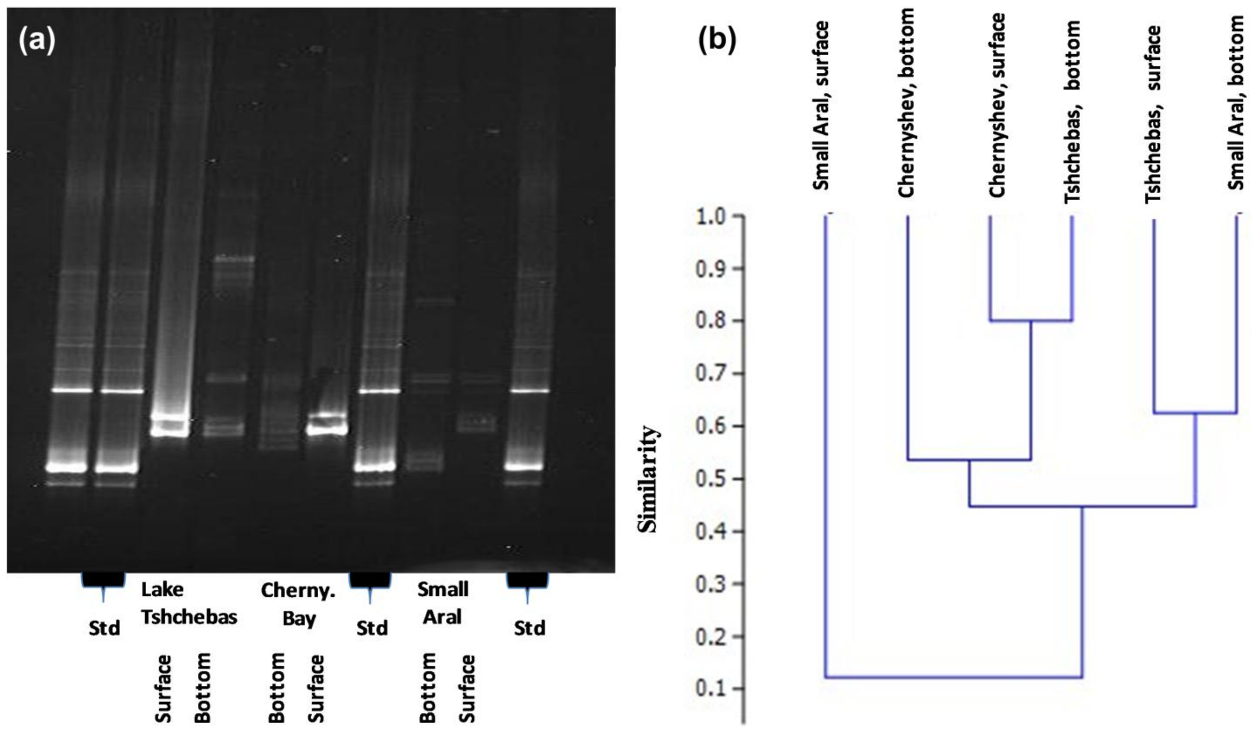

Supplementary Fig. S2. (a) DGGE analysis of Archaea communities in surface and bottom samples of the 3 sampled Aral basins: Lake Tshchebas, Chernyshev Bay (Large Aral Sea), Small Aral Sea; Std: standard. (b) Cluster analysis of DGGE banding pattern.

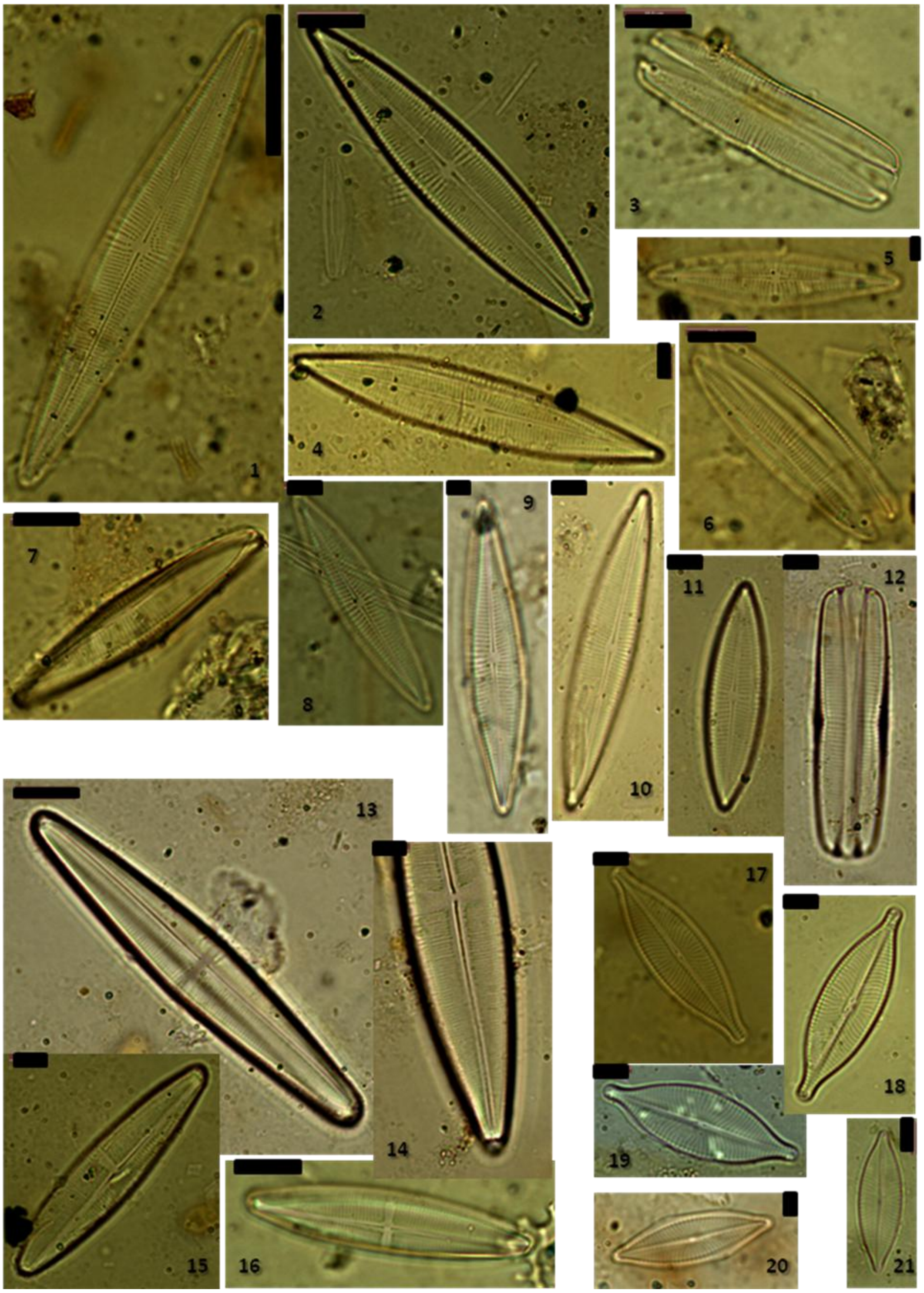

Supplementary Fig. S3. Mass and common species of diatoms from the Small Aral Sea taxocene group (frustles): 1-12 - *Parlibellus cruciculoides*, 13-16 – *Stauroneis salina*, 17-21 – *Navicula salinarum*. Scales: 20 mkm – 1; 10 mkm – 2, 3, 6, 7, 13, 16; 5 mkm – 4, 8, 10, 11, 12, 14, 15, 17, 18, 19, 21; 2 mkm – 9, 20.

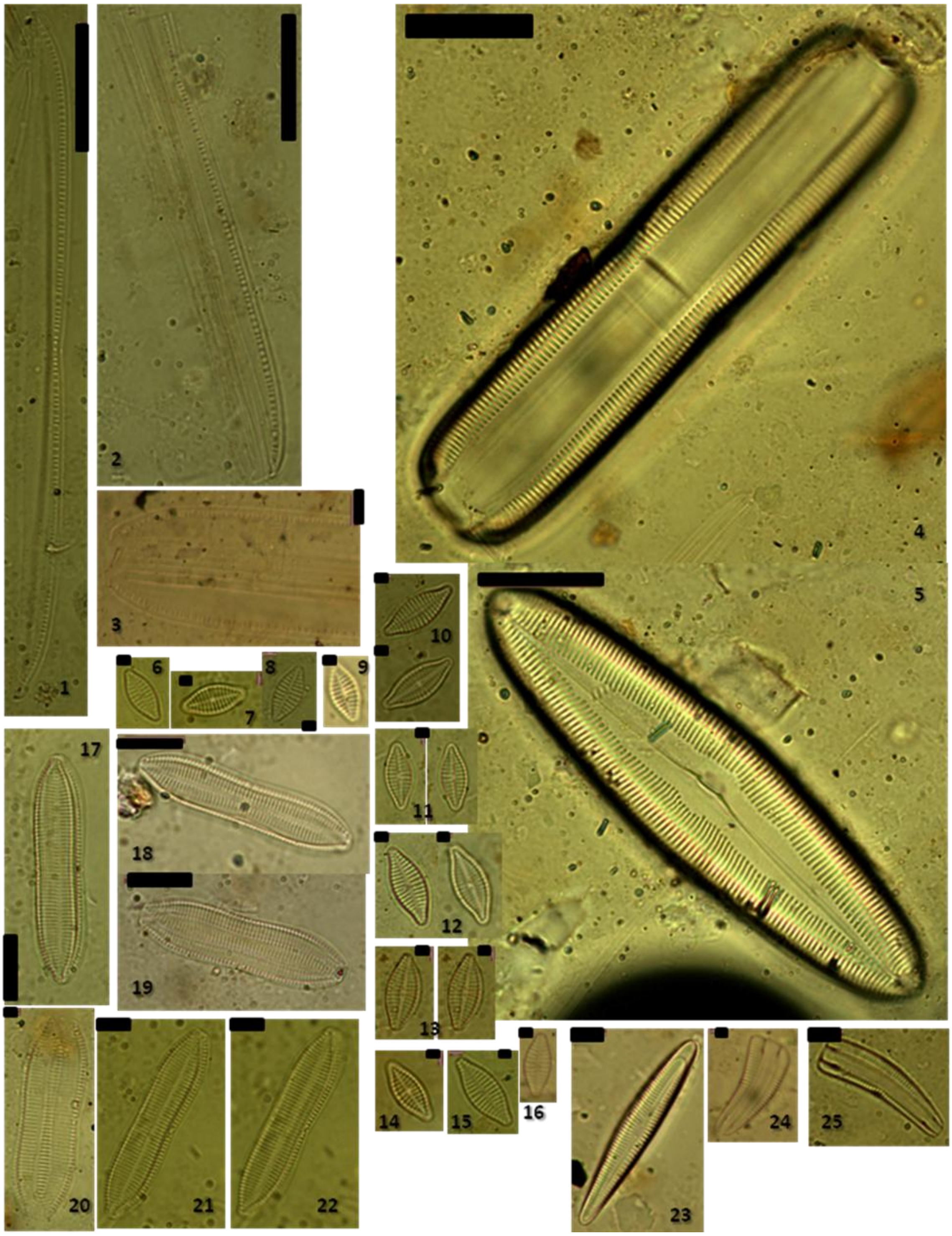

Supplementary Fig. S4. Mass and common species of diatoms from the Small Aral Sea taxocene group (frustles): 1-3 – *Nitzschia lagunae*, 4, 5 – *Pinnularia rupestris*, 6-16 – *Planothidium engelbrechtii*, 17-22 – *Tryblionella apiculata*, 23-25 – *Rhoicosphenia abbreviata*. Scales: 20 mkm – 1, 2, 4, 5; 10 mkm – 17, 18, 19; 5 mkm – 3, 21, 22, 23, 25; 2 mkm – 6-16, 20, 24.

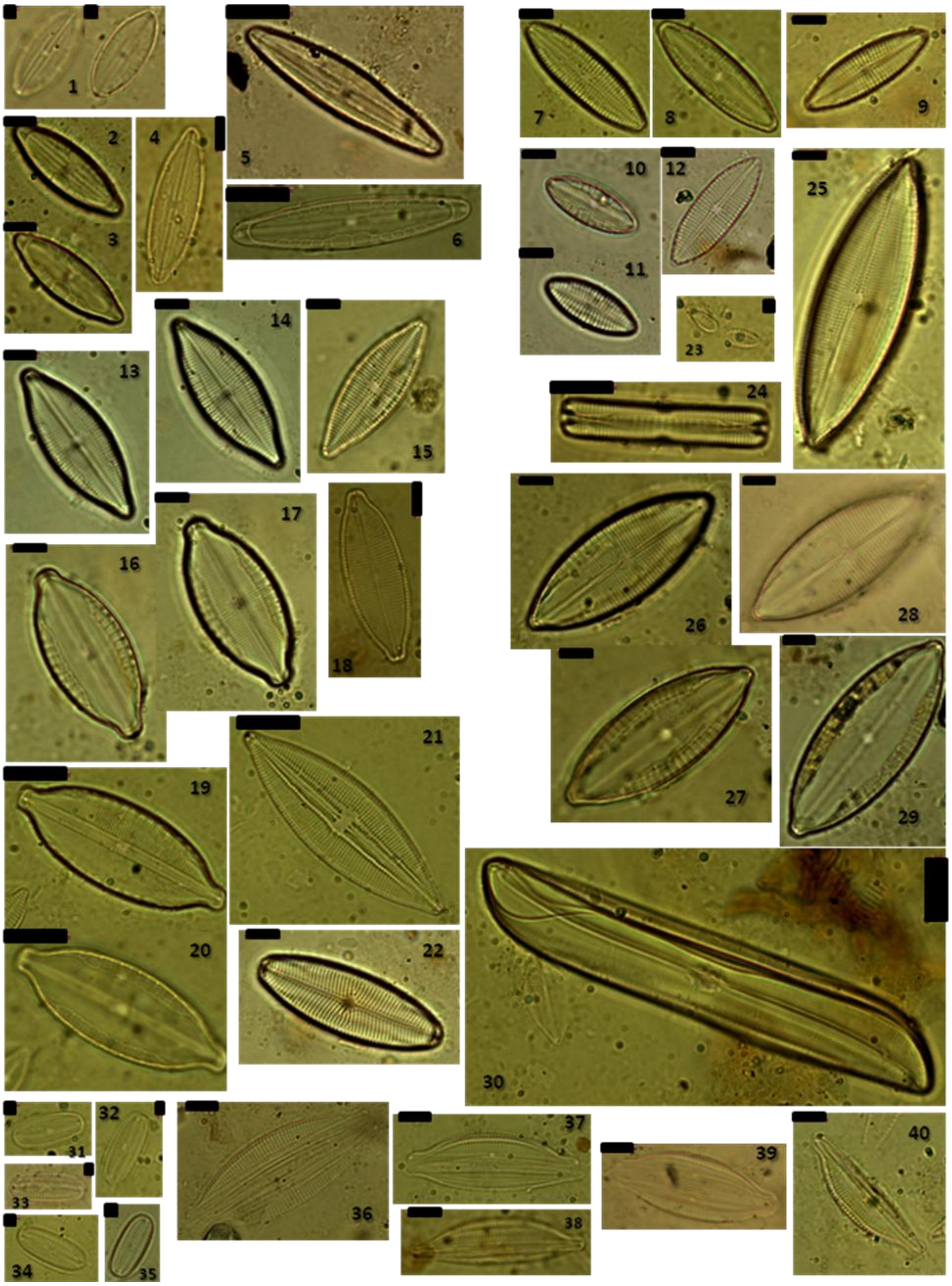

Supplementary Fig. S5. Mass and common species of diatoms from the Small Aral Sea taxocene group (frustles): 1-6 – *Mastogloia pumila*, 7-12 – *Mastogloia pusilla*, 13-15 – *Mastogloia smithii*, 16-20 – *Mastogloia baltica*, 21 – *Mastogloia belaensis*, 22 – *Mastogloia elliptica* var. *densei*, 23 – *Martyana atomus*, 24-29 – *Mastogloia lanceolata*, 30 - *Gyrosigma fenestratum*, 31-35 – *Fallacia tenera*, 36-38, 40 – *Halamphora tumida*, 39 – *Amphora holsaticoides*. Scales: 10 mkm – 5, 6, 19, 20, 21, 24, 30; 5 mkm – 2- 4, 7-18, 22, 25-29, 36-40; 2 mkm – 1, 23, 31-35.

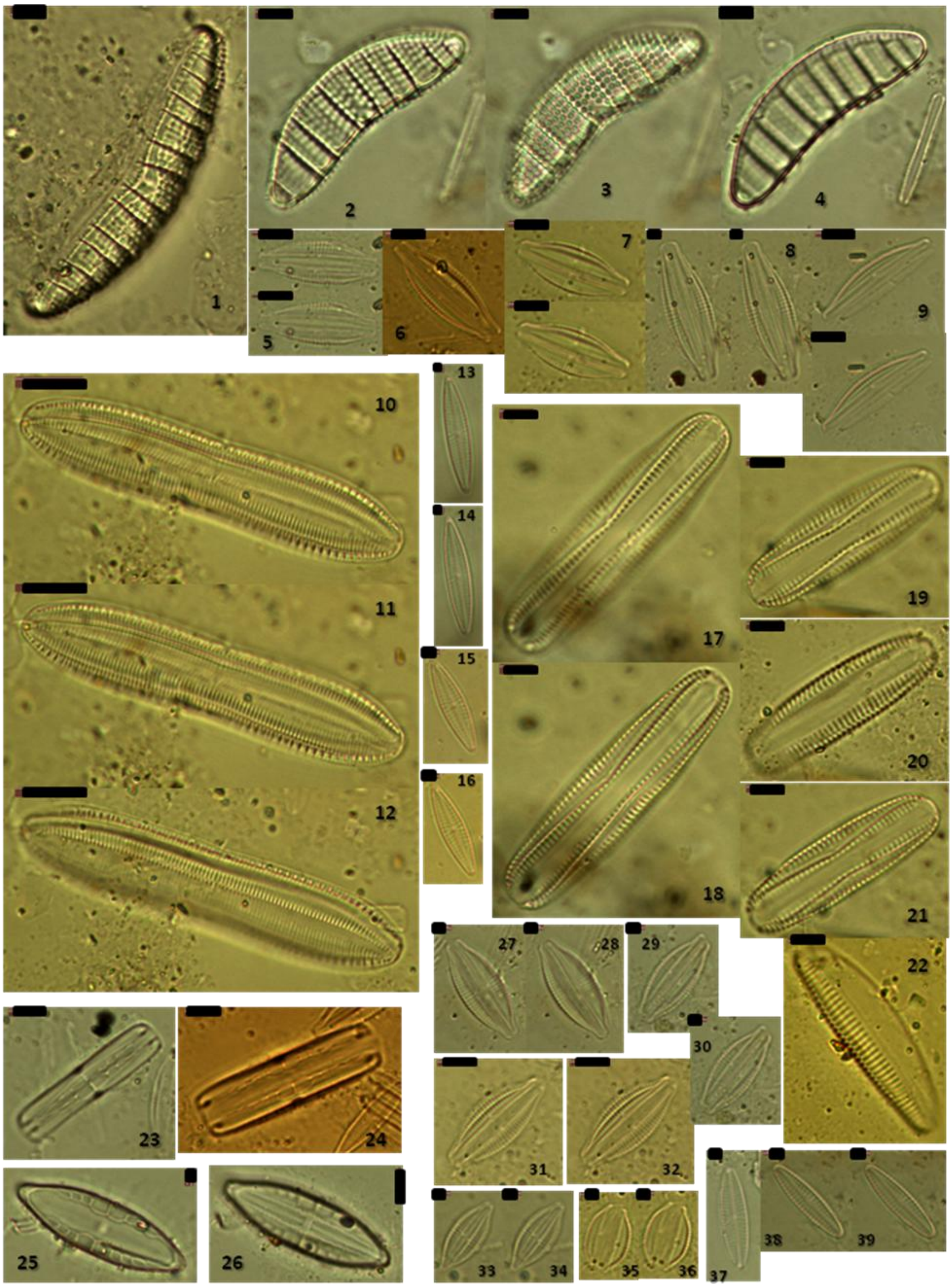

Supplementary Fig. S6. Mass and common species of diatoms from Tshchebas Lake taxocene group (frustles): 1-4 – *Epithemia adnata*, 5-9, 27-32 – *Halamphora acutiuscula*, 10-12 – *Tryblionella hungarica*, 13-16 – *Navicula cryptotenella*, 17-22 – *Amphora pusio*, 23-26 – *Mastogloia pumila*, 33-36 – *Halamphora borealis*, 37-39 – *Navicula* sp. 1 Scale: 10 mkm – 10-12; 5 mkm – 1-7, 9, 17-24, 26, 31, 32; 2 mkm – 8, 15, 16, 25, 27-30, 33-39; 1 mkm – 13, 14.

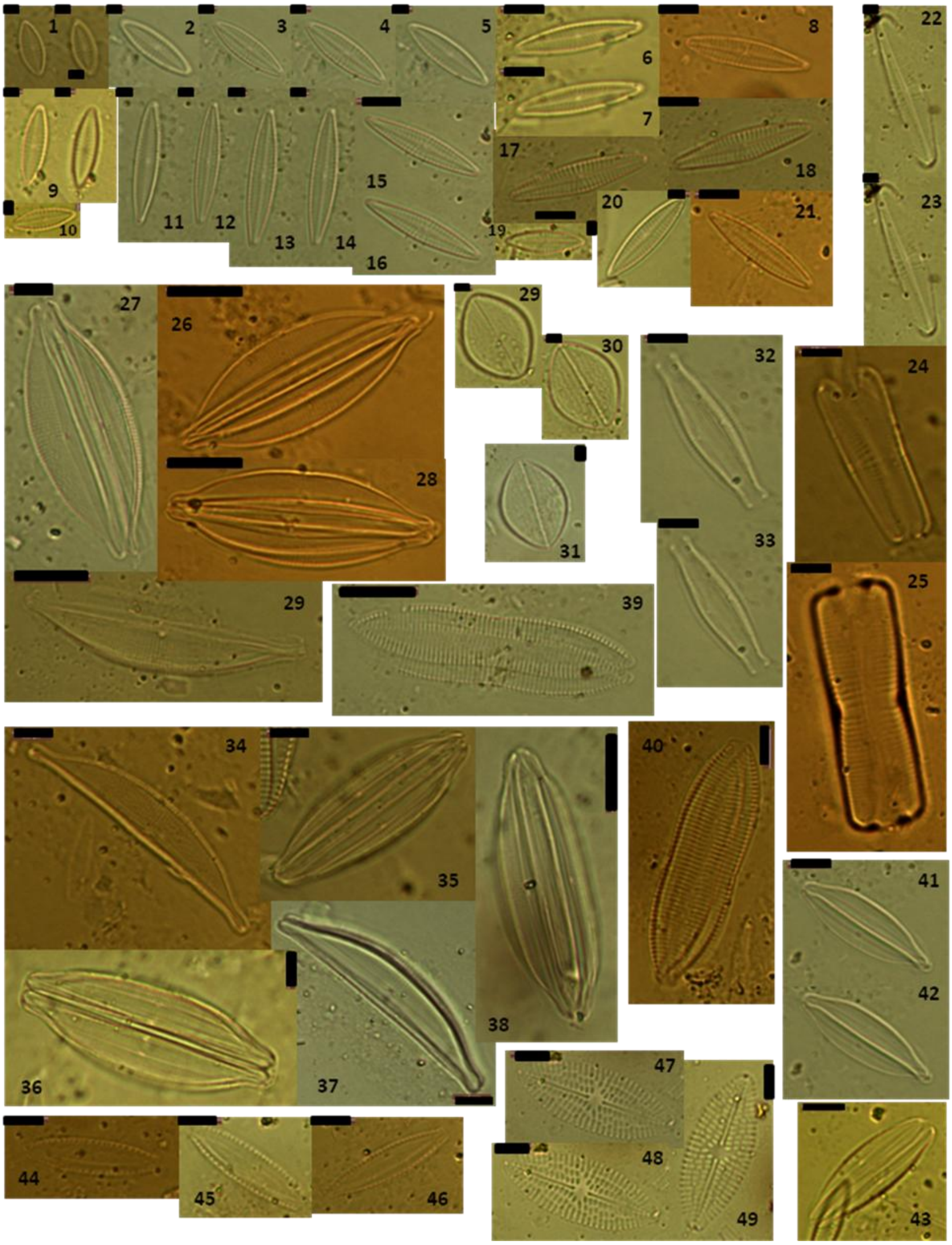

Supplementary Fig. S7. Mass and common species of diatoms from Tshchebas Lake taxocene group (frustles): 1-21 – *Navicula cryptotenella*, 22-25 – *Stauronella* sp. 1, 26-29 - *Halamphora subholsatica*, 29-31 – *Cocconeis placentula* var. *euglypta*, 32-33 – *Halamphora dusenii*, 34-38 – *Amphora holsaticoides*, 39, 40 – *Tryblionella apiculata*, 41-43 – *Halamphora normanii*, 44-46 – *Nitzschia liebetruthii* var. *major*, 47-49 – *Aneumastus* sp. 1 (*A.* aff *minor*). Scale: 10 mkm – 26, 28, 29, 38, 39; 5 mkm – 6-8, 15-18, 21, 24, 25, 27, 32-36, 41-49; 2 mkm – 1-5, 9-14, 19, 20, 22, 23, 29-31.

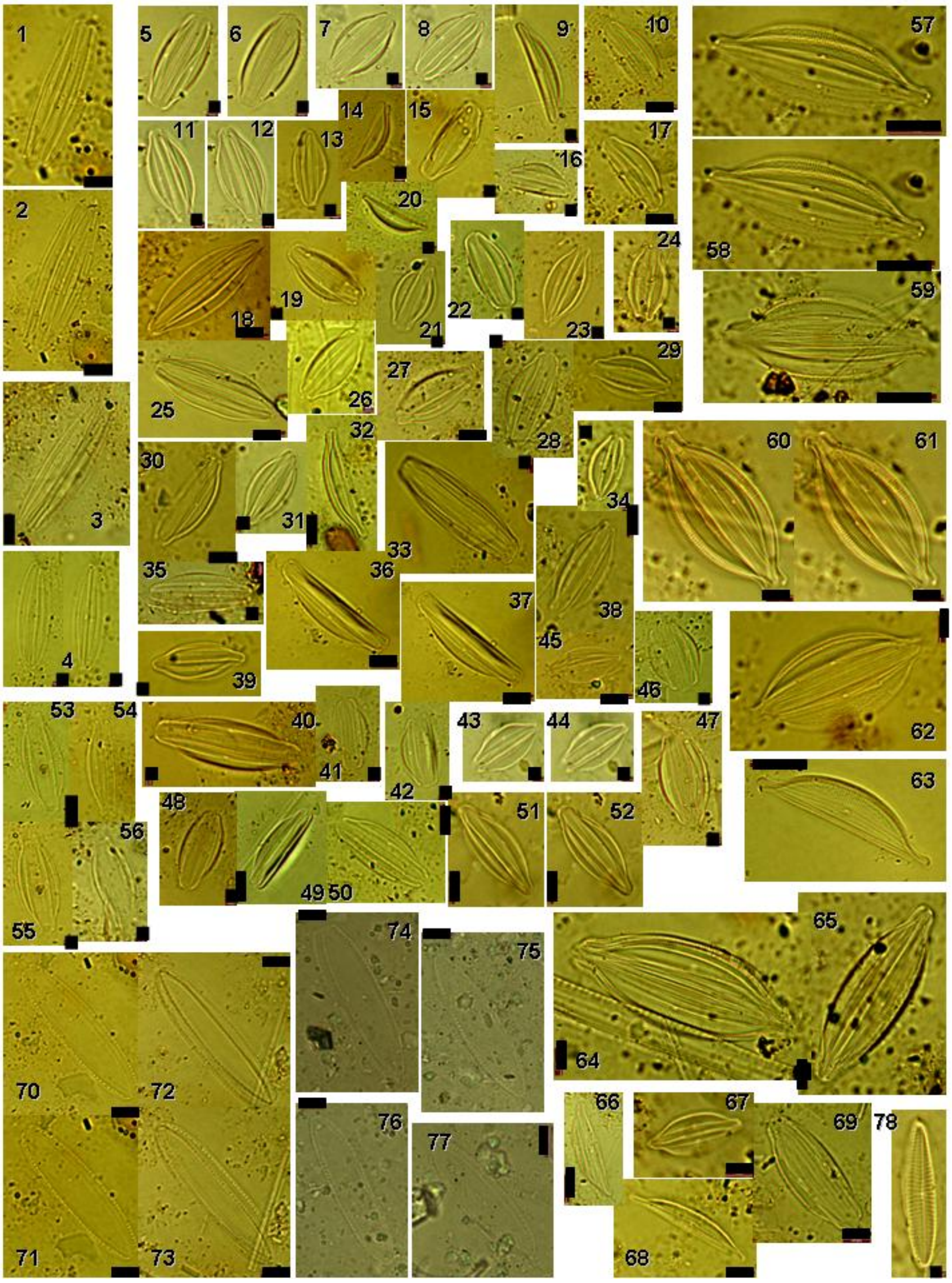

Supplementary Fig. S8. Mass and common species of diatoms from the Large Aral Sea taxocene group: 1-3 *Brachysira styriaca*, 4 – *Brachysira zellensis*, 5-52 – *Halamphora normanii*, 53-56 – *Halamphora acutiuscula*, 57-63 – *Halamphora cymbifera*, 64,65 – *Amphora holsaticoides*, 66-69 – *Amphora pseudoholsatica*, 70-77 – *Nitzschia communis*, 78 – *Navicula radiosafallax*. Scales: 10 mkm – 57-59, 63; 5 mkm - 1-3, 10, 17, 18, 25, 27, 29-32, 36, 37, 45, 49-53, 60-62, 64-69, 70-77; 2 mkm – 4-9, 11-16, 19-24, 26, 28, 31, 33-35, 39-44, 46-48, 55, 56, 78.

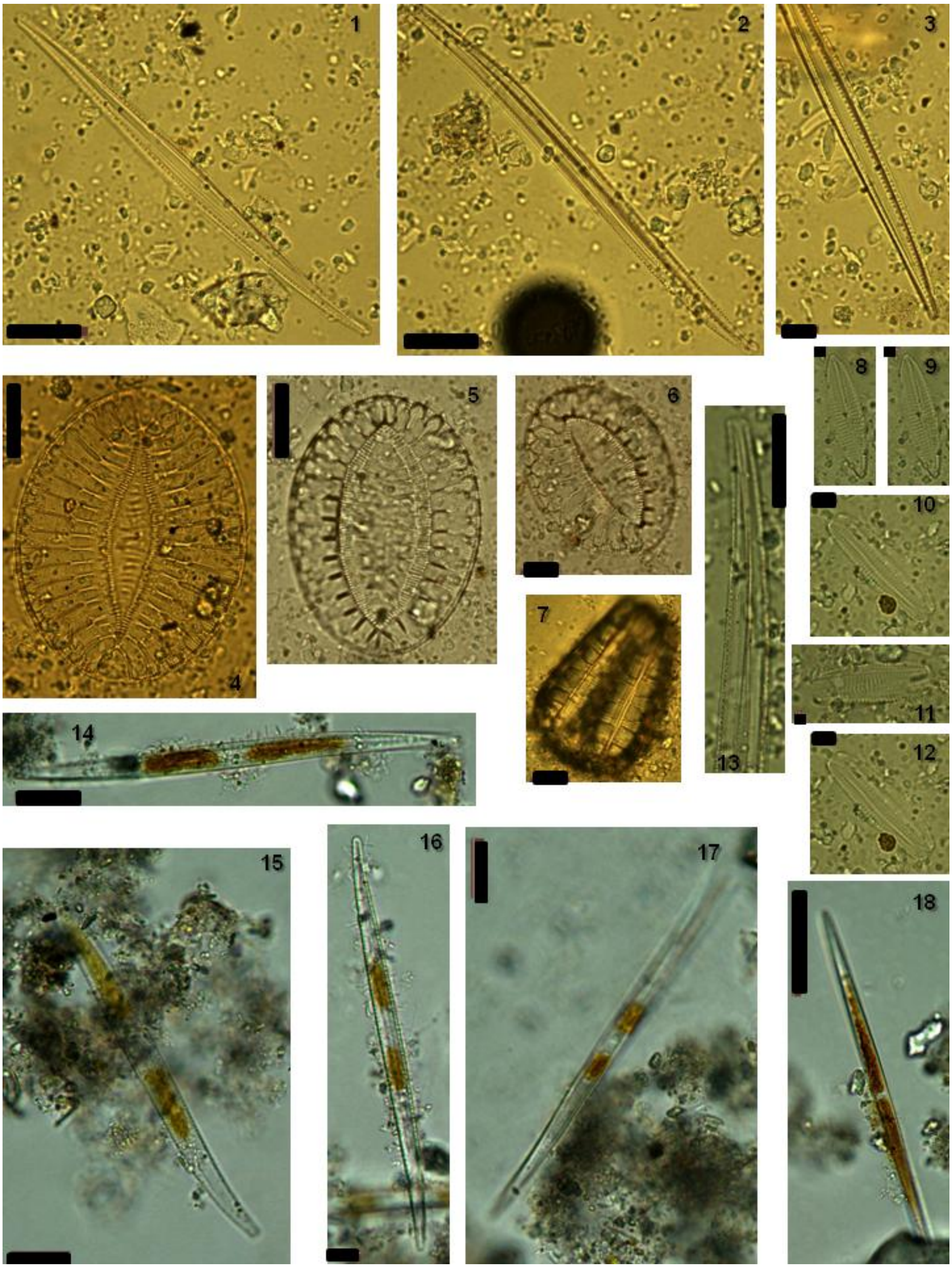

Supplementary Fig. S9. Mass and common species of diatoms from the Large Aral Sea taxocene group (frustles and living cells): 1-3, 13-18 – *Nitzschia sigmaformis* (1-3, 13 – frustles, 14-18 – living cells), 4-7 – *Surirella fastuosa*, 8-12 – *Navicula radiosafallax*. Scales: 20 mkm – 1, 2, 4, 5, 13, 14, 18; 10 mkm – 6, 7, 15, 17 ; 5 mkm – 3, 10, 12, 16; 2 mkm – 8, 9, 11.

Supplementary Table S1. Microphytobenthos samples from the Aral Sea, collected in October 2014. Basins are referred to as: WB – Western Basin of the Large Aral Sea, ChB –Chernyshov Bay, TL – Tshchebas Lake, and SAS – the Small Aral Sea.

№	Water basin	The name of the sample	Coordinates	Depth, m	Ground	Mineralization, ppt	T, ^{0}C	Data
1	WB	WAS850smSILT	N45°4'16,26" E58°23'7,37"	8,5	Silt	115,4	14,4	06.10.2014
2	WB	WAS5smCLAY	N45°5'34,22" E58°20'25,35"	0,05	Clay	115,4	15,2	07.10.2014
3	WB	WAS20smCLAY	N45°5'21,44" E58°20'10,71"	0,2	Clay	115,4	15,0	07.10.2014
4	WB	WAS0smCLAY2	N45°5'21,96" E58°20'9,28"	0	Clay	115,4	15,2	07.10.2014
5	WB	WAS0smCLAY1	N45°5'34,30" E58°20'24,85"	0	Clay	115,4	15,2	07.10.2014
6	WB	WAS5smCLAY1	N45°6'2,67" E58°21'0,73"	0,05	Clay	115,4	15,2	08.10.2014
7	WB	WAS5smSTONE2	N45°5'52,90" E58°20'48,52"	0,05	Stone	115,4	15,2	08.10.2014
8	WB	WAS5smSAND1	N45°6'8,11" E58°21'1,27"	0,05	Sand	115,4	15,2	08.10.2014
9	WB	WAS5smSTONE1	N45°5'41,06" E58°20'38,32"	0,05	Stone	115,4	15,2	08.10.2014
10	WB	WAS0smSHELL	N45°5'36,13" E58°20'29,48"	0	Shell	115,4	15,2	08.10.2014
11	ChB	WASChB0sm SAND	N45°49'19,56" E59°13'33,32"	0	Sand	115,4	11,8	22.10.2014
12	TL	TL0smONFIL	N46°19'17,66" E59°40'20,61"	0	Surface of filamentous algae	91,9	10,3	23.10.2014
13	TL	TL20smSAND	N46°19'17,52" E59°40'20,75"	0,2	Sand	91,9	10,3	23.10.2014
14	TL	TL0smundFIL SAND	N46°19'17,66" E59°40'20,61"	0	Sand under filamentous algae	91,9	10,3	23.10.2014
15	TL	TL30smSAND	N46°19'17,44" E59°40'20,88"	0,3	Sand	91,9	10,3	23.10.2014
16	TL	TL15smSAND	N46°19'17,54" E59°40'20,70"	0,15	Sand	91,9	10,3	23.10.2014
17	SAS	SAS0smOSILT	N46°21'40,85" E60°8'44,00"	0	Organic sludge	11,1	11,6	25.10.2014
18	SAS	SAS5smOSILT	N46°21'43,74" E60°8'44,46"	0,05	Organic sludge	11,1	11,6	25.10.2014
19	SAS	SAS20smOSILT	N46°21'46,63" E60°8'44,77"	0,2	Organic sludge	11,1	11,6	25.10.2014
20	SAS	SAS30smOSILT	N46°21'49,30" E60°8'45,08"	0,3	Organic sludge	11,1	11,6	25.10.2014
21	SAS	SAS1200smSILT	N46°23`48,78' E60°11`23,34"	12	Organic sludge	11,1	11,3	25.10.2014

Supplementary Table S2. Collection of species that characterize diatom community groupings of the Small Aral Sea. Intra-group similarity based on Bray–Curtis dissimilarity index: 22.4%.

Species	Av.Abund	Av.Sim	Sim/SD	Contrib%	Cum.%
Navicula phyllepta	0,08	4,09	1,06	18,26	18,26
Mastogloia smithii	0,05	1,86	0,66	8,28	26,54
Mastogloia pusilla	0,05	1,77	0,62	7,9	34,44
Mastogloia lanceolata	0,04	1,65	0,62	7,36	41,8
Halamphora tumida	0,03	1,3	0,59	5,82	47,62
Planothidium engelbrechtii	0,04	1,29	0,83	5,74	53,36
Parlibellus cruciculoides	0,03	1,28	0,6	5,73	59,1
Navicula salinarum	0,02	1	0,73	4,45	63,54
Pinnularia rupestris	0,02	0,89	0,62	3,95	67,49
Mastogloia pumila	0,04	0,73	0,34	3,24	70,73
Tryblionella hungarica	0,03	0,7	0,41	3,13	73,86
Tryblionella apiculata	0,04	0,54	0,64	2,41	76,27
Gyrosigma acuminatum	0,01	0,44	0,5	1,96	78,23
Rhoicosphenia abbreviata	0,02	0,43	0,52	1,93	80,16
Pleurosigma salinarum	0,01	0,43	0,51	1,9	82,06
Fallacia tenera	0,02	0,41	0,39	1,83	83,88
Amphora holsaticoides	0,01	0,35	0,41	1,55	85,44
Nitzschia lagunae	0,01	0,35	0,41	1,55	86,99
Mastogloia baltica	0,01	0,23	0,9	1,04	88,03
Seminavis strigosa	0,01	0,19	1,14	0,83	88,86
Amphora commutata	0	0,17	1,15	0,76	89,62
Stauroneis salina	0,02	0,17	1,15	0,76	90,38

Supplementary Table S3. Collection of species that characterize diatom community groupings of Tshchebas Lake. Intra-group similarity based on Bray–Curtis dissimilarity index: 45.67%.

Species	Av.Abund	Av.Sim	Sim/SD	Contrib%	Cum.%
Halamphora acutiuscula	0,14	9,18	1,5	20,11	20,11
Navicula cryptotenella	0,14	7,11	0,83	15,57	35,68
Halamphora dusenii	0,07	5,81	5,52	12,72	48,4
Amphora holsaticoides	0,08	5,61	1,14	12,28	60,67
Cocconeis placentula var. *euglypta*	0,09	4,56	1,11	9,99	70,66
Halamphora cymbifera	0,05	2,86	1,13	6,26	76,92
Mastogloia pumila	0,08	2,61	0,68	5,71	82,63
Amphora pusio	0,06	1,99	0,53	4,35	86,98
Nitzschia liebetruthii var. *major*	0,04	1,92	0,84	4,2	91,18

Supplementary Table S4. Collection of species that characterize diatom community groupings of the Large Aral Sea. Intra-group similarity based on Bray–Curtis dissimilarity index: 42.13%.

Species	Av.Abund	Av.Sim	Sim/SD	Contrib%	Cum.%
Nitzschia communis	0,26	16,24	1,67	38,54	38,54
Nitzschia sigmaformis	0,25	14,39	1,17	34,16	72,7
Halamphora normanii	0,12	4,23	0,49	10,03	82,73
Halamphora cymbifera	0,08	2,53	0,59	6	88,73
Navicula radosafallax	0,06	1,86	0,56	4,43	93,15

Supplementary Table S 5. Diatom taxocene diversity (Shannon-Wiener index, (H`)), Pielou's evenness index (J’) and the probability of interspecific encounter (PIE - Gini–Simpson index) for the Aral Sea basins.

Sample	H`	J`	PIE
WAS850smSILT	2,069	0,833	0,832
WAS5smCLAY	1,220	0,880	0,671
WAS20smCLAY	1,088	0,785	0,604
WAS0smCLAY2	0,921	0,839	0,539
WAS0smCLAY1	1,784	0,812	0,775
WAS5smCLAY1	1,156	0,718	0,583
WAS5smSTONE2	1,644	0,845	0,767
WAS5smSAND1	1,393	0,778	0,691
WAS5smSTONE1	1,844	0,839	0,810
WAS0smSHELL	1,784	0,917	0,809
WASChB0smSAND	1,733	0,967	0,813
TL0smONFIL	1,956	0,941	0,845
TL20smSAND	2,166	0,941	0,867
TL0smundFILSAND	2,607	0,920	0,911
TL30smSAND	2,659	0,873	0,908
TL15smSAND	2,094	0,843	0,848
SAS0smOSILT	2,796	0,869	0,924
SAS5smOSILT	2,776	0,781	0,917
SAS20smOSILT	3,908	0,811	0,962
SAS30smOSILT	3,424	0,798	0,952
SAS1200smSILT	2,120	0,921	0,858
Basin	H` mean	J` mean	PIE mean
Large Aral Sea	1,512	0,837	0,718
Tshchebas Lake	2,297	0,904	0,876
Small Aral Sea	3,005	0,836	0,923

Supplementary Table S 6. Summary of primary characteristics observed in the basins of the Aral Sea in October, 2014. H` – Shannon-Wiener index of diatom taxocene diversity.

Basin	*Salinity maximum, g/kg*	*Stratification*	*Surface level*	*H`*	*Biodiversity*
Western Large Aral	121.5	strong	falling	1.5	low
Chernyshev bay	> 133	extremely strong			
Lake Tshchebas	92	slightly stratified	fluctuating	2.3	moderate
Small Aral (Shevchenko bay)	11	mixed	relatively stable	3	most diverse
